# Static Aerated Composting of African Swine Fever Virus-Infected Swine Carcasses with Rice Hulls and Sawdust

**DOI:** 10.3390/pathogens12050721

**Published:** 2023-05-16

**Authors:** Mark Hutchinson, Hoang Minh Duc, Gary A. Flory, Pham Hong Ngan, Hoang Minh Son, Tran Thi Khanh Hoa, Nguyen Thi Lan, Dale W. Rozeboom, Marta D. Remmenga, Matthew Vuolo, Robert Miknis, Lori P. Miller, Amira Burns, Renée Flory

**Affiliations:** 1 Maine Food and Agriculture Center, University of Maine Cooperative Extension, Orono, ME 04473, USA; mhutch@maine.edu; 2 Department of Veterinary Public Health, Faculty of Veterinary Medicine, Vietnam National University of Agriculture Trau Quy, Gia Lam, Hanoi 12400, Vietnam; 3 G.A. Flory Consulting, Mt. Crawford, VA 22841, USA; 4 Department of Anatomy and Histology, Faculty of Veterinary Medicine, Vietnam National University of Agriculture, Trau Quy, Gia Lam, Hanoi 12400, Vietnam; 5 Department of Pathology, Faculty of Veterinary Medicine, Vietnam National University of Agriculture, Trau Quy, Gia Lam, Hanoi 12400, Vietnam; 6 Department of Animal Science, Michigan State University, East Lansing, MI 48824, USA; 7 Center for Epidemiolgy and Animal Health, Veterinary Service, Animal and Plant Health Inspection Services, U.S. Department of Agriculture, Fort Collins, CO 80526, USA; 8 Department of Statistics, Colorado State University, Fort Collins, CO 80523, USA; 9 English Department, Johns Hopkins University, Baltimore, MD 21218, USA

**Keywords:** carcass management, African Swine Fever virus, compost, inactivation

## Abstract

Identifying and ensuring the inactivation of the African Swine Fever virus in deadstock is a gap in the swine industry’s knowledge and response capabilities. The results of our study demonstrate that ASFv in deadstock was inactivated using static aerated composting as the carcass disposal method. Replicated compost piles with whole market hogs and two different carbon sources were constructed. In-situ bags containing ASFv-infected spleen tissue were placed alongside each of the carcasses and throughout the pile. The bags were extracted at days 0, 1, 3, 7, 14, 28, 56, and 144 for ASFv detection and isolation. Real-time PCR results showed that DNA of ASFv was detected in all samples tested on day 28. The virus concentration identified through virus isolation was found to be below the detection limit by day 3 in rice hulls and by day 7 in sawdust. Given the slope of the decay, near-zero concentration with 99.9% confidence occurred at 5.0 days in rice hulls and at 6.4 days in sawdust. Additionally, the result of virus isolation also showed that the virus in bone marrow samples collected at 28 days was inactivated.

## 1. Introduction

African Swine Fever virus (ASFv) is a major threat to swine producers, and the worldwide prevalence of ASFv has significantly impacted the socioeconomics of the global swine industry and trade [1]. The highly infectious disease caused by ASFv poses challenges in production systems, and economically devastating depopulation measures can be required to stop the disease’s spread [2]. The current US Department of Agriculture (USDA) protocol is to “stamp out” the virus with the controlled euthanasia of infected herds. Proper biosecure disposal of carcasses is required to prevent further spread.

Composting is an effective and economical method of carcass management [3,4,5]. Compost is a biologically active process involving many different types of nonpathogenic bacteria, fungi, and actinomycetes decomposing organic materials [6,7]. These organisms compete with pathogenic organisms for resources and create metabolic heat. The competition for resources, along with the heat, are important factors for the inactivation of pathogenic organisms [8]. Previous studies demonstrated that composting was able to inactivate dangerous viruses, including the foot and mouth disease virus (FMDv) [5] and the porcine epidemic diarrhea (PED) virus [9].

Appropriate selection of carbon materials, known as bulking agents, is essential to ensure the success of composting. Each candidate has different characteristics, such as the carbon-to-nitrogen (C:N) ratio, cellulose and lignin concentration, nutrient concentration, porosity, and water-holding capacity [6,7,8]. The selected bulking agents affect the C:N ratio, pH, microorganisms, porosity, aeration, moisture, and temperature of compost piles, thereby influencing the efficacy of the composting process, especially the inactivation of pathogens and the decomposition of carcasses [8,10].

Vietnam is one of the world leaders in the production of rice and wood. Rice hulls and sawdust are abundant in Vietnam, though they are not always effectively recycled. In a previous study, rice hulls have been shown to be effective carbon material for composting ASFv-infected swine carcasses in warm weather; however, the availability of rice hulls is seasonal and local [11]. The evaluation of other potential carbon materials is needed to ensure an adequate supply of bulking agents during an ASF outbreak, regardless of the season. The aim of this study is to compare the efficacy of rice hulls and sawdust in the process of composting ASFv-infected swine carcasses in cool weather.

## 2. Materials and Methods

### 2.1. The Construction of Compost Piles

**Figure 1:**
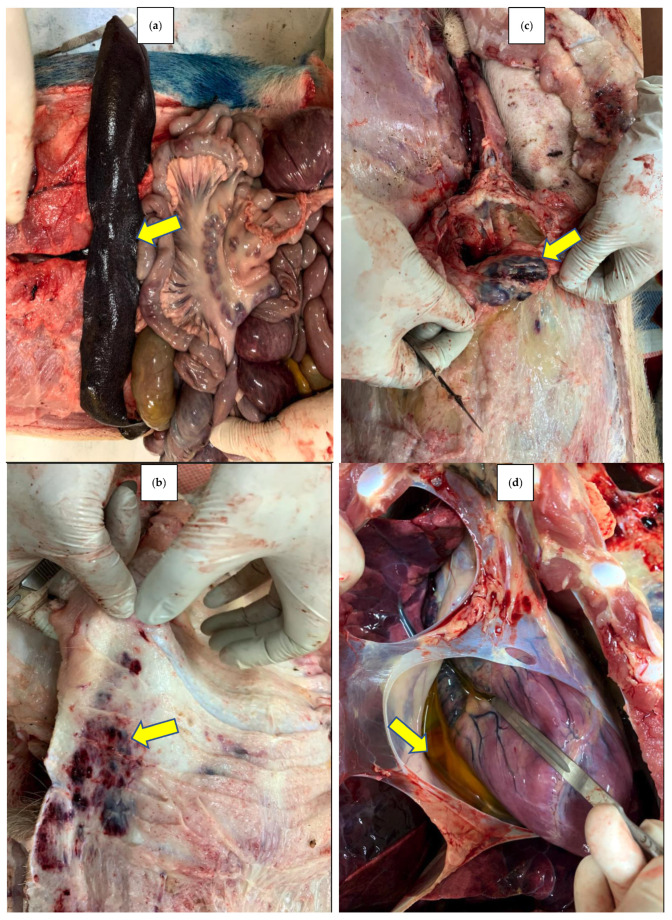
Pathology of the selected swine carcasses used for composting. Enlarged and hemorrhagic spleen (**a**) and lymph nodes (**b**). Necrotic lesions on/under the skin of the abdomen (**c**). Excess of yellow fluid in the heart (**d**).

This study used a randomized complete block design structure with 3 replications of two different carbon materials. The depth within the carbon material type created a split-plot treatment structure with repeated measures over time. A total of 6 market-size hogs with typical ASFv symptoms were used in this study. Prior to composting, the carcasses were diagnosed with ASF by necropsy, and lymph nodes were also collected from each of the pigs for Real-time PCR (qPCR) to confirm the presence of ASFv (Figure 1).

**Figure 2:**
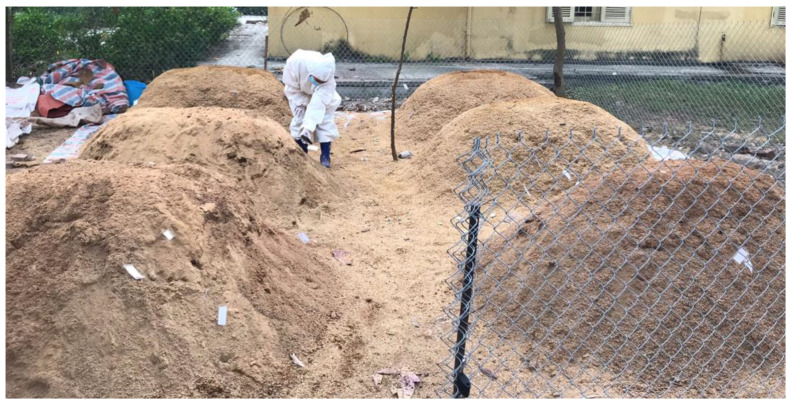
Static aerated swine carcass compost piles in this study.

At the Veterinary Hospital of the Vietnam National University of Agriculture (VNUA), 6 static, aerated compost piles were constructed according to the USDA Large Animal Protocols [12] (Figure 2).

**Figure 3:**
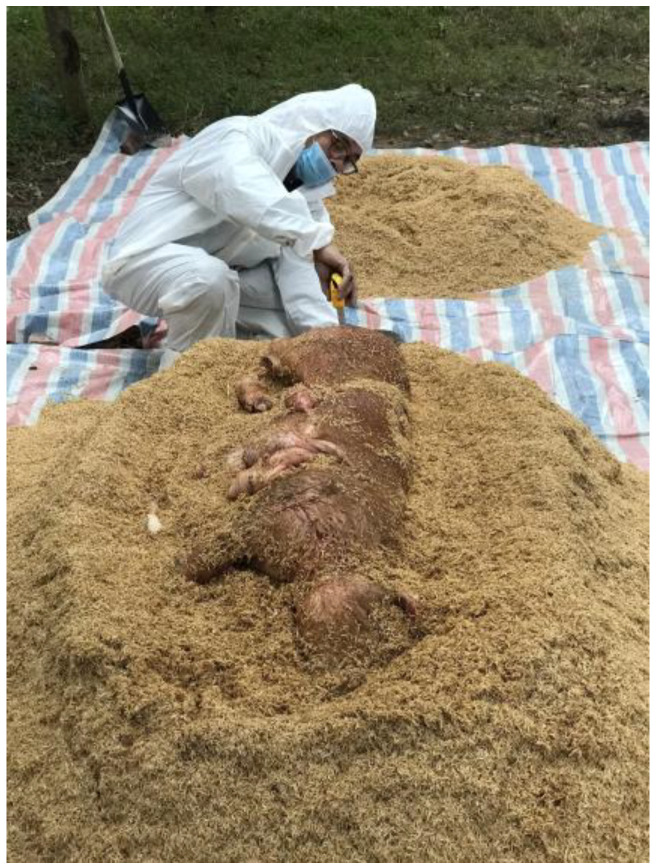
Placement of market-size hog on compost base material of rice hulls.

**Figure 4:**
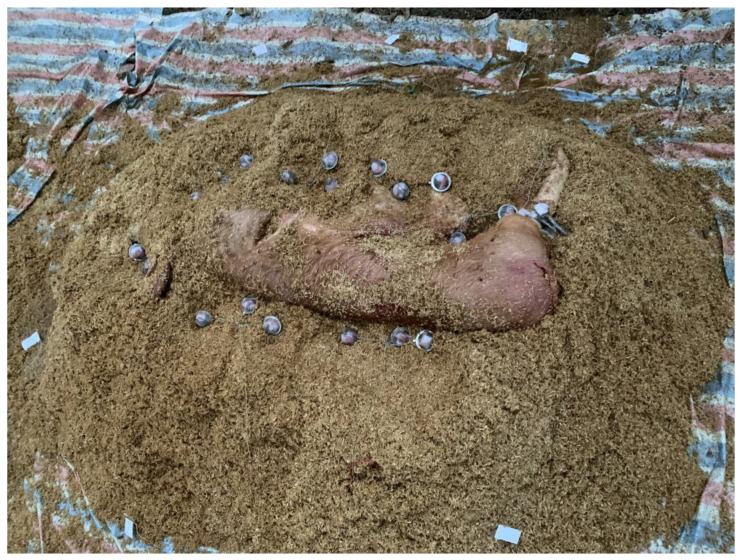
Placement of sample bags around the carcass.

Each compost pile was composed of approximately 7–8 cubic yards of carbon material (rice hulls or sawdust) with 1 infected hog carcass of 60–80 kg. Carbon material (40 cm) was used to form the base of each compost pile. Next, an infected pig carcass was placed on top of the carbon layer (Figure 3). The first set of 21 Dacron in situ bags (Ankom, NY, USA) containing spleen tissue and 3 Dacron bags containing femur bone was then placed around the carcass at the pile core (Figure 4). The carcass was then covered with 30 cm of carbon material, either rice hulls or sawdust and the second set of Dacron bags (21 bags) was placed at this level, in the pile top. In this study, each sample bag was placed in a tea strainer attached to a metal cord to facilitate retrieval. Lastly, 30 cm of carbon material was added on top of the second set of Dacron bags. The entire compost pile was covered with approximately 3.0–4.0 cubic yards of carbon material.

The data logger leads were placed at 2 depths (pile core and pile top) to monitor the temperature near each set of Dacron bags. Temperatures of the compost piles and air temperatures were also monitored daily by a handheld thermometer.

### 2.2. Sample Preparation

At days 0, 1, 3, 7, 14, 28, 56, and 144, Dacron bags containing spleen tissue (3 bags for each day, except day 0, with only 1 bag) were extracted from each compost pile to determine the survival of ASFv. At days 0 and 28, femur bone samples were withdrawn from the compost pile for analysis. Each sample was thoroughly homogenized in Phosphate Buffered Saline (PBS) using Retsch MM400 (Retsch, Dusseldorf, Germany). After, the homogenate was centrifuged at 3000 rpm for 10 min, and the supernatant was collected for DNA extraction and virus isolation.

### 2.3. Detection of African Swine Fever Virus

The DNA of ASFv was extracted using a MagMAX™ Viral/Pathogen Nucleic Acid Isolation Kit (Thermo Fisher, Waltham, MA, USA) following the manufacturer’s instructions. qPCR was carried out following the method previously described by Tignon et al. [13] using a TaqMan probe (5′-FAM-TTCCATCAAAGTTCTGCAGCTCTT-TAMRA-3′) and primer pair (Forward, 5′-TGCTCATGGTATCAATCTTATCG-3′; Reverse, 5′-CCACTGGGTTGGTATTCCTC-3′). The mixture (25 µL) of PCR reagents contained 5 µL of nuclease-free water, 12.5 µL of PCR master mix 2X (Invitrogen superscript III qRT-PCR, Thermo Fisher, USA), 2.5 µL of the primer-probe mix (forward primer (0.6 µM), a reverse primer (0.6 µM), a TaqMan probe (0.3 µM)), and 5 µL of DNA template. QPCR samples were analyzed on a CFX96 qPCR system under the following conditions: 1 cycle at 95 °C for 2 min, 45 cycles at 95 °C for 15 s, and 60 °C for 45 s.

### 2.4. Isolation of African Swine Fever Virus

**Figure 5:**
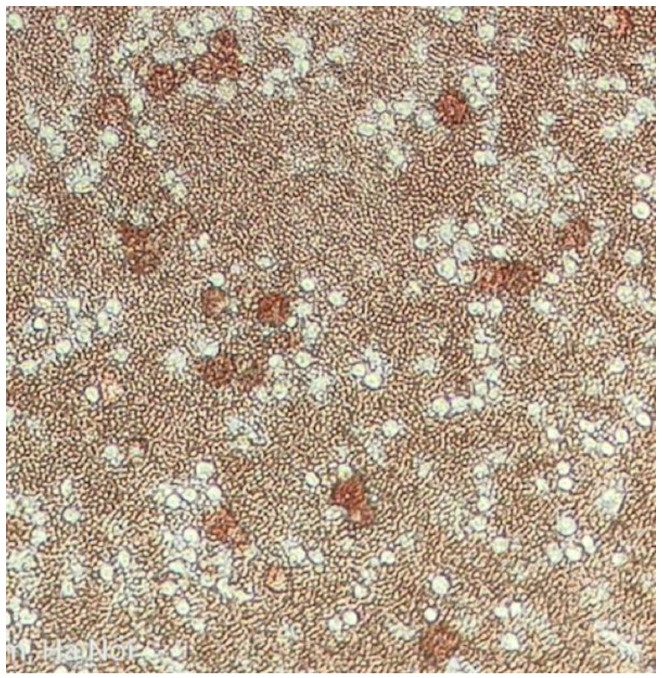
Haemadsorption in ASF virus-infected cells. Arrow indicates HAD rosettes.

Virus isolation was performed by liquefying frozen Porcine Alveolar Macrophages (PAMs) in a water bath at 37 °C. The cell suspension was centrifuged at 2000 rpm for 10 min at room temperature. After centrifugation, the supernatant was removed, and the pellet was washed with 5 mL of PBS buffer. The washed pellet was mixed with 10 mL of culture medium (RPMI 1640, 10% Fetal bovine serum) to reach the cell level of 5 × 10^6^ cells/mL. An aliquot of cell suspension was pipetted into a flat bottom microplate (24 wells). The plate was then incubated for 16–24 h at 37 °C in 5% CO_2_. Following the incubation, PAMs were mixed with 100 µL of 10-fold serial dilutions of the prepared sample (4 wells for each dilution). The mixture was incubated for 30 min at the same conditions as mentioned above. Wells were not inoculated with the sample for negative controls, while haemadsorbing ASFv was used as a positive control. After incubation, the suspension was removed and replaced with 200 µL of fresh RPMI supplemented with 10% of fetal bovine serum, 1% antibiotic and antifungus, and 10% swine erythrocyte. The plate was stored in an incubator at 37 °C in 5% CO_2_. The PAM cells in the plate were observed under a microscope daily for 7 days for the presence of cytopathic effect (CPE) or haemadsorption (HAD) (Figure 5). Virus concentration results are reported as HAD50.

### 2.5. Characteristics of Final Compost

Compost piles were mixed thoroughly before samples were taken for chemical and physical analysis on day 144.

For the determination of the pH value of the compost product, 20 g of the sample was homogenized with 180 mL of deionized water. The mixture was left at room temperature for 30 min before the pH was measured by an SI Analytics Lab 855 pH meter (Xylem Analytics Germany GmbH, Mainz, Germany).

The moisture content was determined following the standard method D2974-13 of the American Society for Testing and Materials International (ASTM International). The collected sample (20 g) was kept in an oven at 110 ± 5 °C for 24 h for drying. The amount of moisture in the sample was equal to the difference in the weight of the sample before and after drying.

To determine the electrical conductivity (EC), the sample (5 g) was homogenized with 50 mL of water. Following homogenization, the sample was left at room temperature for 30 min before it was measured using a Hach HQ14D EC meter (Hach, Ames, IA, USA).

The measurements of the total nitrogen (TN) and total organic carbon (TOC) were performed by the Kjeldahl and Walkley–Black methods. The C:N ratio was then determined based on the carbon and nitrogen content.

### 2.6. Statistical Analysis

The analyses were conducted using R Statistical Software (v.4.1.1, R Core Team 2021). Viral concentrations (log_10_ HAD50) from virus isolation were modeled using a fixed-effects regression with carbon material type, depth of burial, number of days since burial, and the interaction of carbon material type and number of days since burial as factors. On day 0, 6 true replicates were measured, 1 for each carcass. Each compost pile was associated with a specific carcass. After day 0, 3 sub-samples were measured at each combination of extraction day and depth within each pile; each set of 3 sub-samples (also called pseudo-replicates) counted only as 1 true replicate. Additional models that explored random effects for pile number and averaging of sub-samples were run to establish the correct degrees of freedom. The results of these models were extremely similar to the results presented in this paper and are excluded for brevity. The number of days to 1 HAD50 was estimated using calibration methods, and a bootstrap method provided estimates of uncertainty.

After reviewing different methods of managing censored data, day 3 virus concentrations for the rice hull piles were set at the detection limit (10^2^ HAD50), which best represents information from unmasked observations while allowing for uncertainty in the behavior of responses below the detection limit. Virus concentrations for day 7 and beyond were all below the detection limit of the cell culture test; only a negative result from the cell culture test was obtained, and these non-numeric values were not included in the statistical analysis.

There are some important assumptions associated with the statistical methodology. Virus concentration is assumed to decay in a straight line on the log_10_-scale over time. Virus concentration below the detection limit is also assumed to decay in a straight line as a continuation of the model calculated for the data observed above the detection limit. Violation of these assumptions could lead to a bias in the results presented in this paper, particularly if there is a curve in the true virus concentration decay as it approaches zero. The standard assumptions of multiple linear regression models were evaluated and found to be valid. The method of setting the censored data equal to the detection limit of 10^2^ HAD50 artificially reduces the prediction interval widths while increasing the mean time to 1 HAD50. Factors held constant in the study design that could affect the virus decay in real-world scenarios are the weight and age of the pig carcasses, weather conditions, and the quality of the compost pile construction.

## 3. Results

**Figure 6:**
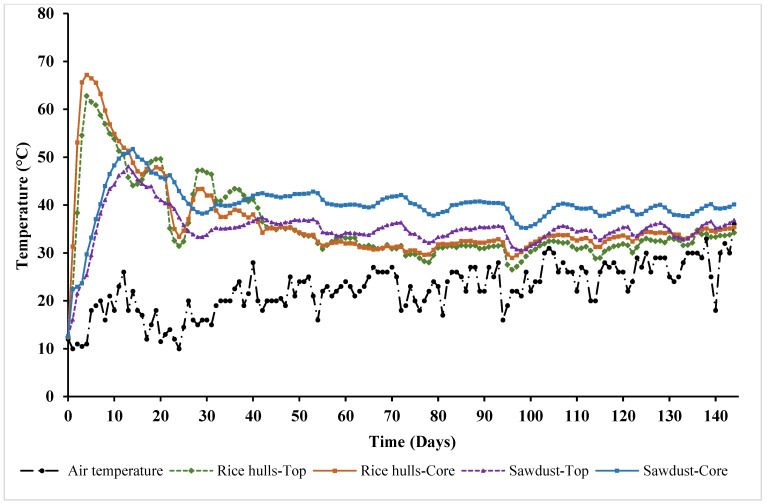
The temperature profile of compost piles with rice hulls and sawdust.

Temperature profiles of compost piles using rice hulls (RH) and sawdust (SD) as carbon materials are shown in Figure 6. For RH, temperatures at the core were higher than those at the top for the first 2 weeks. Core temperatures increased sharply and reached a peak of 67.2 °C on day 4. Similarly, the top temperature also rapidly increased and reached the highest point (62.8 °C) on day 4. Both temperatures exhibited a downward trend after day 4 but remained above 50 °C until day 12. Compared to RH, the temperature of SD was lower during the first 12 days. Figure 6 shows that temperatures of SD rose more slowly but also decreased more slowly compared to those of RH. The core temperature of SD reached a peak of 51.72 °C on day 14, while the highest temperature at the top position was 48.08 °C, observed on day 13. For the first 10 days, temperatures at the top and core of SD were quite similar. However, the temperature at the core remained noticeably higher than that at the top from day 11 to day 144. Regardless of the carbon material, temperatures of the compost piles were much higher than the air temperature throughout the experiment (Figure 6).

The results of qPCR obtained prior to composting showed that all 6 swine carcasses were positive for ASF. At day 0, the Ct values of lymph node samples ranged from 17.75 to 20.99, the Ct values of spleen samples ranged from 16.63 to 20.56, and the Ct values of bone marrow ranged from 16.10 to 21.52. In general, Ct values increased over time, which indicated a decreasing trend in virus concentration; however, DNA of ASFv was still detected in both spleen and bone marrow samples on day 28. Spleen sample Ct values ranged from 21.53 to 32.70, while those of bone marrow samples ranged from 21.87 to 31.05. There was no observed difference between Ct values obtained from sawdust and rice hulls, nor between core and top positions, on day 28.

Virus isolation revealed that the initial concentration of ASFv at day 0 for spleen and bone marrow ranged from 10^6^ to 10^6.7^ HAD50 and from 10^5.3^ to 10^6.3^ HAD50, respectively. The virus concentration quickly reduced below the detection limit (10^2^ HAD50) of virus isolation after 3 days of composting for the rice hulls experiment and after 7 days for the sawdust experiment. Virus isolation also demonstrated that the virus in bone marrow samples collected on day 28 was not infectious.

**Figure 7:**
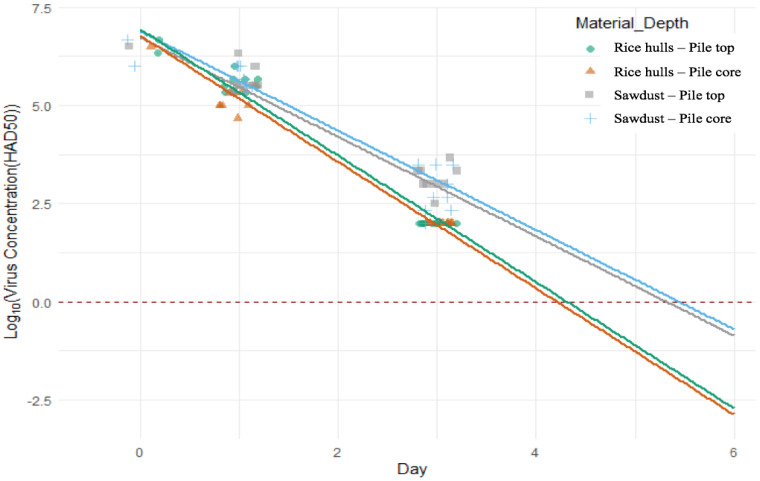
The prediction of virus concentration during compost.

The fitted lines were extrapolated past the range of observable data to visualize when this model predicts total virus decay at a concentration of 1 HAD50 (Figure 7 and Table 1). The mean virus concentration in the population for sawdust at the pile core is estimated to decay to 1 HAD50 in 5.3 days, with 999 out of 1000 pigs reaching 1 HAD50 by 6.3 days. The mean virus concentration in the population for sawdust at the pile top is estimated to decay to 1 HAD50 in 5.4 days, with 999 out of 1000 pigs reaching 1 HAD50 by 6.4 days—slightly longer than at the core. The decay for rice hulls was a little more than one day faster, with the mean virus concentration in the population estimated to decay to 1 HAD50 in 4.2 days at the core and 4.3 days at the top. The predicted time to decay for individual pigs for rice hulls shows that 999 out of 1000 pigs reached 1 HAD50 by 4.9 days at the pile core and 5.0 days at the top (Figure 7 and Table 1).

Total N, total C, C:N ratio, pH, and EC of samples collected from RH on day 144 were 2.57, 25.7, 10, 7.59, and 2.53, respectively (Table 2). The total N, C, C:N ratio, pH, and EC of those extracted from SD were 1.66, 30.01, 18, 6.93, and 1.68, respectively (Table 2).

**Figure 8:**
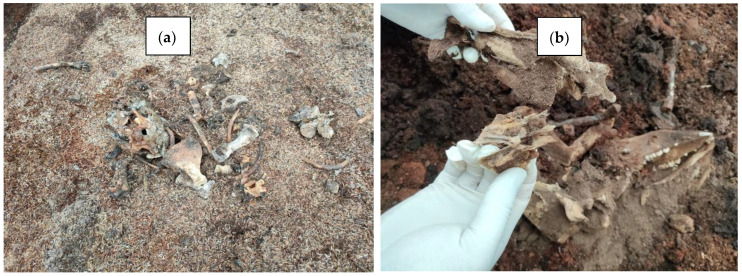
Bones remaining at day 144 of composting market-size hogs with rice hulls (**a**) and sawdust (**b**).

The excavation of compost piles at day 144 showed that whole carcasses were almost completely degraded in both RH and SD. The carcass’s soft tissue was entirely decomposed, with only large bones remaining visable, most of which had become spongy in texture (Figure 8a,b).

## 4. Discussion

The results of this study suggest that composting is an effective measure to inactivate ASFv in swine carcasses during cool weather. The mechanisms for ASFv inactivation during composting in this study are not fully understood. They may relate to several factors, including temperature, pH, moisture content, C:N ratio, oxygen, metabolic by-products, enzymes produced by indigenous microflora, and predation by native microorganisms. The inactivation of ASFv may also depend on the virus strains and the compost formulation [14]. For aerobic composting, high temperatures generated by indigenous microbial activity are usually considered the main factor contributing to pathogen inactivation [7,15]. In the present study, ASFv was inactivated on day 3 for RH with temperatures of 54.54 °C at the top and 65.63 °C at the core. For SD, ASFv was found to be inactivated on day 7, with temperatures of 38.19 °C and 40.18 °C at the top and core, respectively. This result is in agreement with previous studies about virus inactivation during composting. The research of Guan et al. [4] on the survival of Avian Influenza (AI) and Newcastle Disease (ND) viruses in composting showed that temperatures of 50 °C–55 °C were sufficient to inactivate both AI and ND viruses. Another study conducted by Elving et al. [3] indicated that the AI virus was destroyed at both mesophilic (35 °C) and thermophilic (45 °C and 55 °C) phases of compost. Also, Guan et al. [16] reported that the bovine viral diarrhea (BVD) virus was destroyed at 41 °C when composted. Similarly, a study by Vitosh-Sillman et al. [6] demonstrated that composting could inactivate the PED virus within 24 h at 37 °C. Moreover, an in vitro trial performed by Plowright and Parker [17] indicated that ASFv was heat sensitive. Approximately a 5 log reduction of ASFv was observed after 90 min of treatment at 56 °C. Another in vitro study by Turner and Williams [18] revealed that ASFv was destroyed in 30 min at 50 °C, in 90 s at 56 °C, and within 30 s at 60 °C; therefore, temperature may play a part in ASFv inactivation in our study. However, temperature is not the only factor resulting in pathogen inactivation during composting. A study by Droffner and Brinton [19] investigating the survival of *Salmonella* and *E. coli* in a food waste compost showed that these pathogens were not inactivated when held at temperatures of 60–70 °C for more than 9 days but were inactivated when the temperature declined from 62 °C to 40 °C in the compost curing, suggesting that other factors may affect the survival of *Salmonella*. Larney and Hao [20] examined the survival of total coliforms in cow manure compost using wood chips or straw as bulking agents. The initial level of total coliforms in both compost types was 7.86 log CFU/g. The concentration was then reduced to 3.38 log CFU/g by 7 days and 1.69 log CFU/g by 14 days. Temperatures of wood chip and straw compost reached a peak of 66.7 °C and 68.78 °C at 45 and 23 days, respectively. Thus, the decrease in the coliform population may result from factors (physical, chemical, biological) beyond temperature.

Microbial antagonism could be another factor involving ASFv inactivation in compost. The survival of ASFv may be affected by indigenous microorganisms present in compost feedstocks, as they may compete with ASFv for nutrients, receptors, and hosts. Predation may also influence the survival of ASFv in compost. For example, the study by Puri and Dudley [21] suggests that predation by protists was the cause of the decrease of *E. coli* O157:H7 in compost.

The pH may also contribute to ASFv inactivation in compost. The results of a previous study showed that the addition of 1% of NaOH or Ca(OH)_2_ led to ASFv inactivation within 150 s at 4 °C, while the activation slowed to 30 min with the addition of 0.5% of NaOH or Ca(OH)_2_. The addition of NaOH or Ca(OH)_2_ at 1% did not have an effect on the survival of swine vesicular disease virus at 22 °C after 30 min; however, the addition of 1.5% of NaOH or Ca(OH)_2_ resulted in the inactivation of the virus at both 4 °C and 22 °C [18].

Volatile acids were suggested to be another mechanism for virus inactivation [22]. The lipophilic end of the organic acid can interact with the lipid membrane of the enveloped virus, resulting in virus inactivation. In addition, organic acids have a negative effect on the nucleic acid and capsid proteins of non-enveloped viruses, preventing virus adhesion to host cells [23]. Ammonia produced by the ammonification of proteins during composting may also be able to inactivate viruses, as cell membranes, viral envelopes, and capsids are permeable to ammonia [24].

The selection of bulking agents for compost is vital, as they can produce some chemical compounds that directly affect pathogen inactivation. In addition, bulking agents may indirectly influence pathogen inactivation through the C:N ratio, pH, native microorganisms, porosity, aeration, and moisture of compost piles [9]. In the present study, ASFv was inactivated faster in RH (3 days) compared to SD (7 days). There are various means of explaining this result. Rice hulls have a higher porosity than sawdust; consequently, RH has better aeration, stronger aerobic microbial activities, and higher temperature than SD. Aeration is known to be essential for microbial proliferation, heat production, and gas volatilization in composting [25]. Therefore, aeration is an important factor contributing to pathogen inactivation [26]. Previous studies showed that aerobic decomposition was more efficient in eradicating viruses such as enteric viruses and naked ssDNA viruses compared to the anaerobic process [27,28]. It has been reported that the presence of oxygen helps to reduce by more than half the time the pathogen inactivation in slurry and manures [27,29]. Also, aeration facilitates the proliferation of innate microorganisms present in compost feedstocks that can compete or act as predators of pathogens in compost [30]. Another explanation for the slower inactivation of ASFv using sawdust is the presence of natural compounds, such as polyphenols, which can inhibit aerobic microbial activities [31]. Alternatively, a study by Erickson et al. [32] suggested that phenolics produced during the degradation of lignin influenced pathogen inactivation in compost. In the present study, the volume of materials was the same between RH and SD, the C:N ratios of RH were expected to be lower than SD since rice hulls have been reported to have a lower total organic carbon but higher nitrogen content than sawdust [33,34]. The low C:N ratio in RH may result in stronger ammonia production, which enhances pathogen inactivation [8]. Our result is consistent with previous studies showing faster *Salmonella* and *L. monocytogenes* inactivation at a C:N ratio of 20 compared to that of 40 [14,35]. The results of our study also indicate that temperatures of SD after reaching a peak reduced more slowly than RH temperatures, indicating sawdust is able to retain heat better than rice hulls. On the contrary, the temperature of RH increased faster than SD. This finding is in agreement with previous studies indicating that the temperature of rice hulls compost was higher for the first 20 days but had a shorter thermophilic phase compared with that of sawdust compost [9]. Overall, the results of this study suggest both rice hulls and sawdust can be used for effectively composting ASFv-infected pigs; however, rice hulls appear to be a better choice for rapid inactivation.

## Figures and Tables

**Table 1:** Prediction of ASFv inactivation.

Material Type/Depth	Mean Number of Days to 1 HAD50	Prediction Interval Confidence (%)	Number of Days to 1 HAD50
Sawdust/Core	5.3	98	4.7–5.9
		99.8	4.4–6.3
Sawdust/Top	5.4	98	4.9–6.1
		99.8	4.5–6.4
Rice Hulls/Core	4.2	98	3.8–4.6
		99.8	3.5–4.9
Rice Hulls/Top	4.3	98	3.9–4.7
		99.8	3.7–5.0

**Table 2:** Physiochemical characteristics of compost product at day 144.

	Rice Hulls	Sawdust
pH	7.59 ± 0.19	6.93 ± 0.31
EC (mS/cm)	2.53 ± 0.19	1.68 ± 0.22
TOC (%)	25.7 ± 1.58	30.01 ± 2.88
TN (%)	2.57 ± 0.26	1.66 ± 0.19
C:N	10	18

## Data Availability

The data that support the findings of this study are available from the corresponding author upon reasonable request.
